# A step in the right direction: Delphi consensus on a UK and Australian paediatric podiatry curriculum

**DOI:** 10.1186/s12909-022-03138-8

**Published:** 2022-02-24

**Authors:** Julie Reay, Cylie Williams, Chris Nester, Stewart C. Morrison

**Affiliations:** 1 Directorate of Allied and Public Health, University of Salford, Room PO49, Brian Blatchford Building, University of Salford, M6 6PU, Manchester, UK; 2 Monash University, School of Primary and Allied Health Care, 47-49 Moorooduc Hwy, Frankston, VIC 3199 Australia; 3 Directorate of Allied and Public Health, University of Salford, Room PO32, Brian Blatchford Building, University of Salford, M6 6PU, Manchester, UK; 4 School of Health Sciences, University of Brighton, Brighton, UK

**Keywords:** Podiatry, Pre-registration, Paediatric, Curriculum

## Abstract

**Background:**

Previous research shows considerable variation in pre-registration paediatric podiatry curricula, and thus the clinical skills realised prior to graduation. Whilst pre-registration training is guided by regulatory bodies, these high level principles only refer briefly to standards in paediatric practice. An estimated 9% of podiatry caseloads in the United Kingdom (UK) and Australia are dedicated to paediatric service provision. Therefore, it is imperative that curricula support the consistent development of paediatric practice enabling newly registered podiatrists to work safely and effectively with children. Given that the global healthcare work force provides unique opportunities to explicitly align international curricula, the aim of this study was to determine the priorities for a UK and Australian binational pre-registration paediatric podiatry curriculum.

**Methods:**

A four round modified Delphi design was employed to ascertain consensus and agreement of a panel of experts with a special interest in paediatrics working in the UK and Australia. Round 1 contained open questions designed to promote diverse responses on the broad topics of lecturer experience and curriculum organisation and delivery. The answers from Round 1 were developed, through content analysis, into a series of statements presented to the panel for agreement in Rounds 2, 3 and 4.

**Results:**

Of the 297 statements generated following Round 1, 183 were accepted and 114 rejected by the end of Round 4. 109 of the accepted statements related to curriculum content. Participants also agreed on areas relating to lecturer experience, clinical education, and assessment of paediatric skills.

**Conclusions:**

This study is the first of its kind to describe elements of a curriculum for pre-registration podiatry training. The recommendations highlight opportunities that education providers can work towards during curriculum design. They also emphasise the collaboration that is needed between professional bodies, clinicians and higher education institutions when defining guidelines and expectations for paediatric specific skills.

**Supplementary Information:**

The online version contains supplementary material available at 10.1186/s12909-022-03138-8.

## Background

Children and young people represent one fifth of the United Kingdom’s (UK) population and nearly one third of the Australian population [1, 2] and clearly the health of this population will affect future healthcare needs of each country. Lower limb pain is the most common paediatric musculoskeletal problem presenting to UK and Australian general practitioners (GPs) [3–5]. These problems occur in up to 6.2% of young people in the UK yearly, and account for 5% of paediatric presentations to Australian GPs [5, 6]. The real prevalence of paediatric musculoskeletal complaints is likely to be higher, as young people may also present directly to allied health professionals where incidence based data is not published. Access to paediatric health services is therefore, undoubtedly, essential and the overall aim is to resolve short term conditions, thus preventing chronicity, and manage long term problems to limit future impact. In the UK/Australia, podiatrists work in the public or private sectors providing care for people of all ages [7, 8]. Up to 9% of podiatry caseloads are devoted to paediatric work where podiatrists partner with families and other health care providers to prevent and support paediatric foot and lower limb conditions [7–9].

High quality podiatry service provision is underpinned by appropriate education. ‘Pre-registration education’ refers to the specific training, usually organised by a higher education institution and lasting between two and four years, that leads to a professional and academic qualification allowing an individual to become a registered podiatrist and able to practice independently. ‘Post-registration education’ refers to continuous professional development and qualifications undertaken as a registered professional. Whilst pre-registration podiatry standards, curricula and expected competencies are informed by documents produced by quality assurance and statutory regulatory bodies, these only refer briefly to competency standards in paediatric podiatry practice [10–15]. This general guidance leaves much open to interpretation and choices made at specific Higher Education Institutions may not have kept pace with developments in practice. It follows that it is important to provide a practice led interpretation of curriculum priorities. Indeed, evidence of variation, both nationally and internationally, in some areas of the paediatric pre-registration podiatry curricula have been demonstrated by Williams et al. [16]. For example, in the UK, whilst consistent curriculum updates are carried out, there are inconsistencies in the expertise of lecturers, course content and the time devoted to topics. With regards to the educational landscape in Australia, the number of hours devoted to specific paediatric content is mostly consistent, however there are inconsistencies in staff background and the time at which paediatric content is introduced during the course. There are also additional international inconsistencies.

Podiatry remains on the skilled profession shortage list to encourage migration to Australia [17]. It is similarly under ongoing scrutiny within the UK with an increasing number of vacancies [18]. Clear processes on how to register and practice as a podiatrist when moving to the UK from Australia (and vice versa) are defined by the Health and Care Professions Council (UK) and Podiatry Board of Australia with mutual recognition of curricula, delivering equivalent skills and qualifications across each country. This highlights the importance of considering a binational UK-Australian curriculum given that global workforce competencies are key to future consistent evidence-based practice and quality care [19].

Inconsistencies in paediatric training can impact newly registered podiatrists and their patients as they may be responsible for the management of children from the day they work independently. Therefore it is essential that this area is explored further. The aim of this study was to determine the priorities for a binational UK and Australian pre-registration paediatric podiatry curriculum.

## Methods

### Overview

The study design was a modified Delphi technique. Consensus and agreement were gained via a four round modified Delphi online survey. This method congregates expert opinion through a series of iterative questionnaires. This approach was chosen due to its validity in collecting and synthesising expert opinion, suitability across geographically dispersed participants, and because it permits complete anonymity between experts, allowing free expression of opinions [20]. Ethical approval was provided by the University of Salford (HSR1920-005).

### Participants

Participants were eligible to take part if they met two inclusion criteria. Firstly, participants had be registered and working in the UK/Australia at the time of the study. Secondly, participants had to be practicing at level 4 or above of the Paediatric Podiatry Clinical Framework [21], as these levels require high levels of autonomy and self-reflection.

### Recruitment

Recruitment was purposive and undertaken via promotion of written and video-recorded information disseminated on social media (Facebook, Twitter) and via email to professional bodies for advertising or paediatric podiatry special interest groups for dissemination to members. Podiatrists expressed an interest through the project lead (JR) and were provided with a link to establish their eligibility to participate and provide informed written consent. All podiatrists (nineteen in total) who met the inclusion criteria were subsequently invited to participate.

### Procedure

All data were collected via ‘Online Surveys’ (Jisc, Bristol UK). Participants were identifiable to the project team via their participant code, but all data were fully anonymised to other participants. Participants were given four weeks to complete Round 1 and two weeks for subsequent rounds. At the start of each round participants received feedback (through the survey) on the consensus or agreement achieved during the previous round.

### Round 1 design

Round 1 contained 75 open questions supported by background information and references, to capture a large breadth of opinions (Appendix 1). Questions were designed by the project team comprising four academics/clinicians (three podiatrists CW [Australia], CN [UK], SM [UK]; one physiotherapist JR [UK]). The team has extensive expertise in higher education and research with a focus on podiatry and paediatrics.

**Fig. 1:**
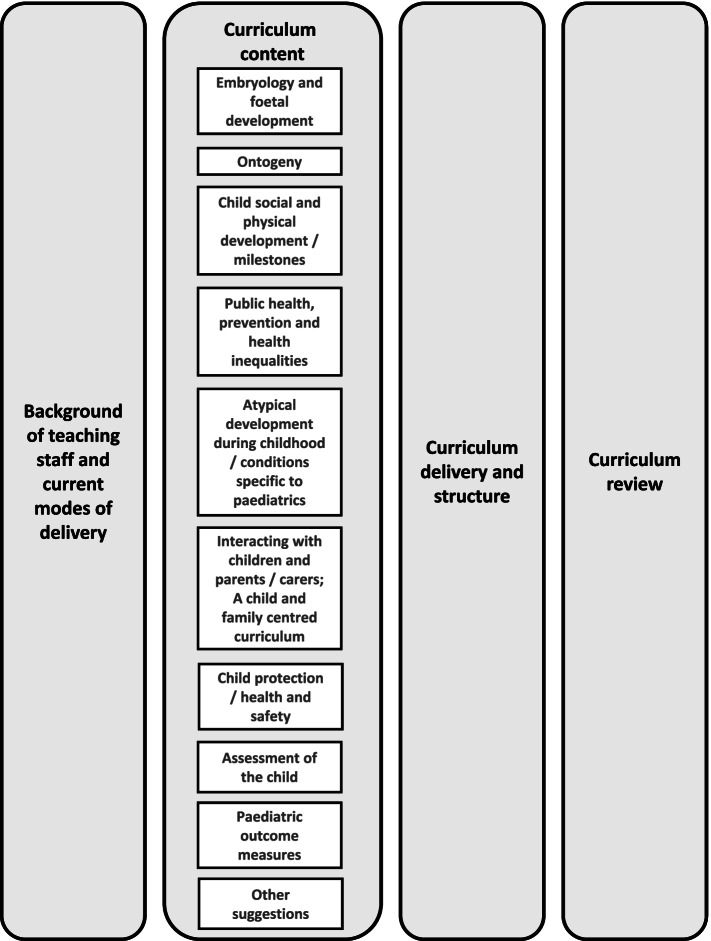
Themes for Round 1 questions

Round 1 questions focussed on common themes identified following a literature review on; podiatry curricula standards; pre-registration standards for healthcare; prevalence of childhood lower limb conditions; podiatry practice standards of competency and proficiency; national healthcare drivers; post-registration paediatric podiatry guidance; and a recent study on podiatry curricula [16]. Round 1 themes are presented in Fig. 1.

### Round 1 analysis

The qualitative open ended response data were exported into Excel 2010 (Microsoft Corp, Redmond Washington) and were analysed (JR, checked by SM) using inductive content analysis to group answers into statements [22]. Ambiguous answers were discussed and a decision made as to how they were incorporated into Round 2 statements (JR and SM). Where 70% (or over) of participants recorded a similar answer it was deemed consensus for that statement [20]. All other answers were formed into closed statements for Round 2.

### Rounds 2, 3 and 4 design

Participants were asked to rate the statements generated through Round 1. A Likert scale was used to gain agreement where: 1 = Very unimportant, 2 = Unimportant, 3 = Neither important nor unimportant, 4 = Quite important, 5 = Very important. Round 2 Likert scale answers were analysed and agreement was achieved when 70% (or more) of participants rated statements as ‘4’ or ‘5’. Statements rated by less than 50% of participants as ‘4’ or ‘5’ were removed. Statements rated by 50-69% of participants as ‘4’ or ‘5’ were put through to Round 3. Rounds 3 and 4 followed the same process as Round 2.

### Subsequent round analysis

The quantitative data from Rounds 2, 3 and 4 were exported from Online Surveys (Jisc, Bristol, UK) into Excel 2010 (Microsoft Corp, Redmond Washington) and agreement for each statement was ascertained using descriptive statistics. It had been agreed *a priori* that the Delphi would close following Round 4, irrespective of consensus and agreement achieved. Where a participant missed a round, they were excluded from subsequent rounds.

## Results

**Fig. 2:**
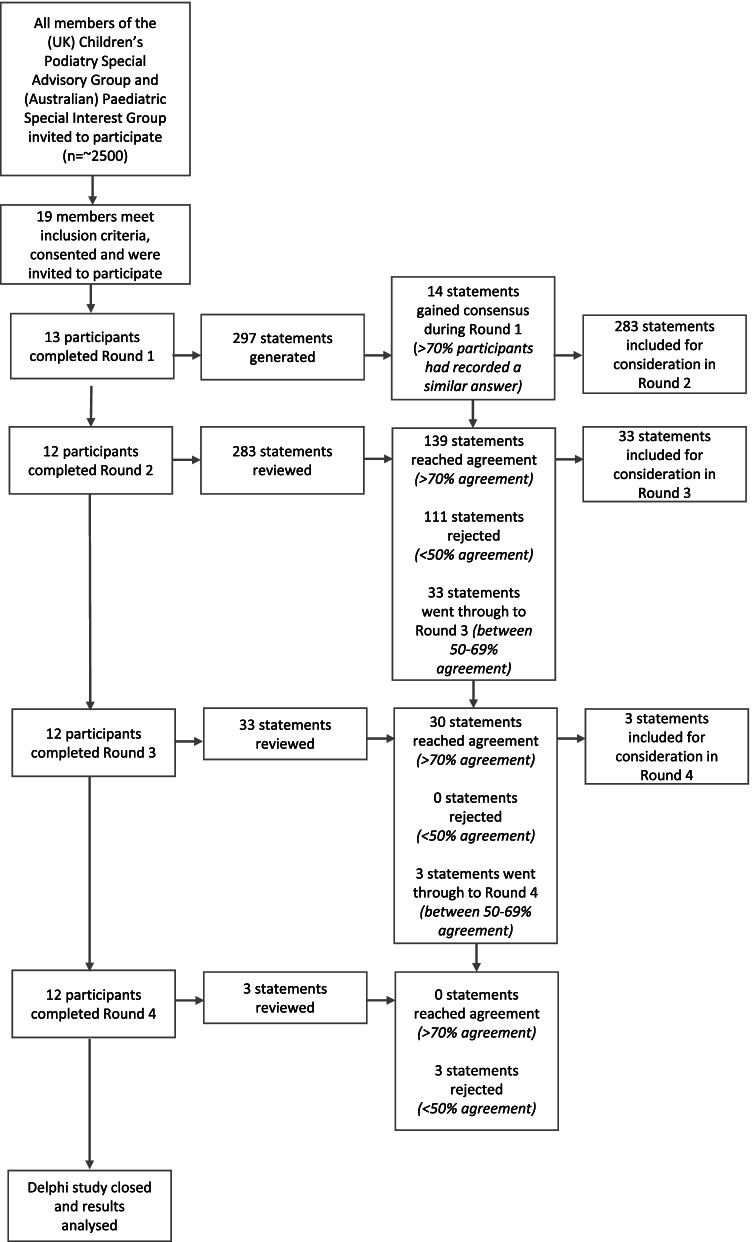
Summary of study process

Figure 2 summarises the study process including number of participants and statements associated with each round. Nineteen participants responded and were invited to participate as all met the inclusion criteria. Of these, 13 participants completed Round 1. One participant failed to respond following Round 1 and was withdrawn. Therefore 12 participants completed all subsequent rounds. Table 1 summarises key participant demographics.

**Table 1:** Participant demographics (based on the 13 participants who contributed to Round 1)

		Frequency (%) of participants [n = 13]
Gender (Female)		7 (54%)
Time since registration as a podiatrist	< 10 years	1 (8%)
	10 years or greater	12 (92%)
Country of qualification	Australia	6 (46%)
	UK	7 (54%)
Highest qualification	PhD	2 (15%)
	MSc or postgraduate qualification	5 (38%)
	Bachelor degree	4 (31%)
	Bachelor degree and currently studying postgraduate qualification, MSc or PhD	2 (16%)
Current country of work	Australia	6 (46%)
	UK	7 (54%)
Current job title	Academic and clinician	2 (15%)
	Clinician	9 (70%)
	Team leader / Head of podiatry	2 (15%)
Primary work setting	Private practice and public sector	4 (31%)
	Public sector	7 (54%)
	Private practice and university	2 (15%)
Percentage of weekly work devoted to paediatrics	Between 20 and 39%	2 (15%)
	Between 40% and 79%	6 (46%)
	Between 80 and 100%	5 (38%)
Paediatric current caseload	< 60%	2 (15%)
	60% or greater	11 (85%)
Contact with podiatry students (hours per month)	0	5 (38%)
	Less than 10	4 (31%)
	10 or greater	4 (31%)
Additional information	Experts reported their involvement in the following roles: Clinical educator, co-authorship of paediatric podiatry framework, delivery of casual workshops for university, provider of training for public sector podiatrists	

Thematic analysis of Round 1 open questions generated 297 statements of which 14 reached consensus during Round 1 (>70% participants responded similarly). Of the 283 statements reviewed during Round 2, 139 reached agreement (>70% rated statement as ‘4’ or ‘5’), 111 were rejected (<50% rated statements as ‘4’ or ‘5’) and 33 were included for consideration in Round 3 (between 50 and 69% rated statements as ‘4’ or ‘5’). Out of the 33 statements progressing to Round 3, 30 reached agreement, none were withdrawn and three progressed to Round 4 (between 50 and 69% of participants rated statements as ‘4’ or a ‘5’). All Round 4 (n=3) statements were rejected. By the end of the Delphi process, 183 (62%) statements were accepted and 114 (38%) rejected.

### Statements relating to overarching themes

#### Background of lecturers / current modes of delivery

Table 2 denotes all of the statements that reached consensus and agreement, whilst Appendix 2 provides a summary of the status of all 297 statements included in the study (whether accepted or rejected). Participants agreed that a lecturer delivering the paediatric curriculum should have a minimum of two years paediatric specific clinical experience.

**Table 2:**
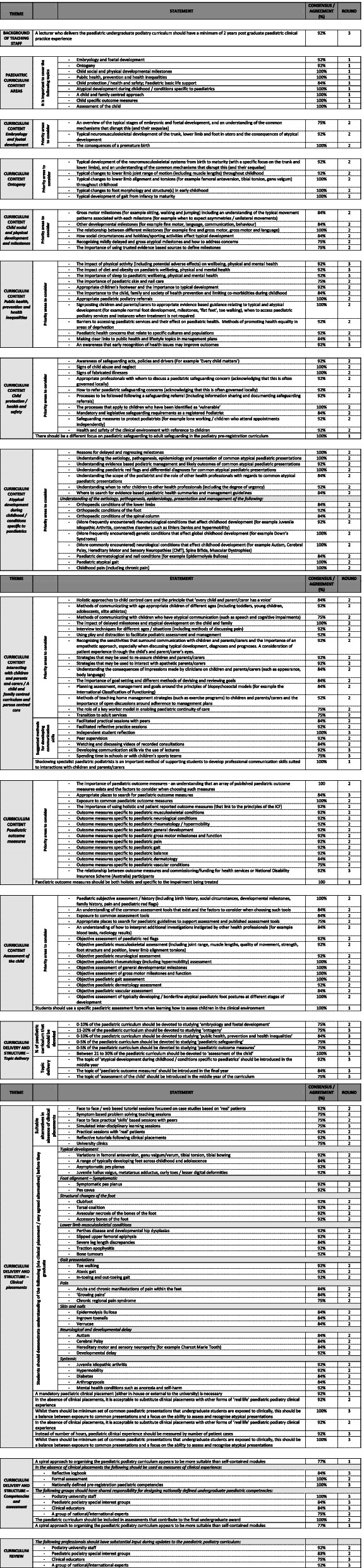
Statements that reached consensus or agreement (All rounds)

#### Curriculum content

During Round 1, consensus was obtained for nine proposed themes appropriate for the curriculum (100% of participants recorded a similar answer for 6/9 themes). Over the course of the Delphi rounds there was agreement on 109/110 (99%) statements relating to curriculum content associated with each of the nine themes. The only statement that did not reach agreement related to the impact of smoking and alcohol on wellbeing, physical and mental health.

#### Curriculum delivery / structure

Few participants responded similarly on how much time should be devoted to curriculum topics, with only 6/38 (16%) statements reaching agreement. Likewise, fewer participants responded similarly on when to introduce topics into programmes, with agreement on 3/45 (7%) statements. However, there was consensus that organising paediatric concepts using a spiral approach [23], where topics are re-visited throughout a course with deepening complexity, was preferable to self-contained modules (77%, Round 1).

A greater number of participants provided similar responses to statements focussing on clinical placements. There was consensus (92%, Round 1) that a mandatory paediatric clinical placement should be embedded in the paediatric podiatry curriculum, but, acknowledged that this is not always possible. It was agreed that it should be acceptable to rely on suitable alternatives (92%), all of which reached agreement (Table 2). No agreement was reached on the minimum hours dedicated to paediatric practice attached to clinical placements. Instead, exposure to a set number of paediatric specific presentations (92%) was suggested but a minimum number of cases could not be defined. It was agreed that this needs to be balanced between exposure to common paediatric podiatry presentations and a focus on the ability to assess and recognise atypical presentations.

Participants agreed that the paediatric curriculum should be included in assessments contributing to the final pre-registration award (100%) and this could be assessed via; a logbook (84% agreement), a nationally defined set of pre-registration paediatric competencies (100%) or formal assessment (100%). There was agreement that competencies should be designed by podiatrists working in higher education (100%), podiatry special interest groups (84%), clinical educators (84%) or national/international experts (75%). Common clinical presentations (33 statements) were identified as key areas that should be understood prior to registration (Table 2).

#### Curriculum review

Agreement was reached on which professional groups should be involved with curriculum review; podiatrists working in higher education (92%); paediatric podiatry special interest groups (83%); clinical educators (75%); and national or international experts (92%). However, there was no agreement on the timing for review of contemporary paediatric practice.

## Discussion

This unique study sought consensus and agreement on key areas within a binational pre-registration paediatric podiatry curriculum. These key areas were determined by a Delphi panel composed of registered podiatrists working in the UK/Australia in the public sector, private practice or academia.

### Background of lecturers

Previous work indicates that paediatric podiatry course content is typically delivered by lecturers with paediatric experience approximately 50% of the time [16]. This is in contrast to the expectations of the experts within this present study who agreed that paediatric curricula should be delivered by lecturers with a minimum of two years paediatric clinical experience. Paediatric practice is a specialist topic area, and, whilst adults and children share some common clinical presentations, managing the care of children requires a specific understanding of physical and psychosocial development, family/carer dynamics, the impact of health conditions on a growing body and conditions specific to childhood [24, 25]. Therefore, in order to successfully facilitate learning in paediatrics, lecturers must have a contemporary and evidence-based knowledge and grounding in the theoretical frameworks of paediatric-centred care, facilitating correct, contextualised, holistic and stimulating learning drawing on lived experience. This is more likely to promote an interest in the subject matter, positively influencing student engagement and learning [26]. Studies in medical education highlight that role modelling, such as excellence in teaching and demonstrating enthusiasm and high standards, plays a key role in the professional development of students [27, 28]. A role model for paediatric podiatry practice surely must be built on similarly solid foundations.

### Curriculum content

There appears to be a mismatch between current practice and the recommendations from the Delphi panel in terms of curriculum content. This is demonstrated by Williams et al. [16], who reported that between 15 and 40% of surveyed universities did not include key topics in their curricula, such as general paediatric conditions, embryology and foetal development. However, in this present study, statements relating to curriculum content reached the most agreement, resulting in a specific set of recommendations. It should be noted that agreed statements in this area represent the panel’s beliefs on what pre-registration students should encounter, as a minimum, in order to develop the skills, knowledge and understanding that underpin safe practice. In light of this, designing a pre-registration curriculum is a balancing act [29, 30]. It is essential that core areas of practice must be covered during training and experience, and this can leave training that relates to special populations, such as paediatrics, competing for space in a busy curriculum [19]. However, whilst this study aimed to gain consensus on the priorities of a binational paediatric podiatry curriculum, it did not aim to limit the number of statements generated or establish the feasibility and practicality of embedding these into a curriculum.

Many of the statements in Table 2 are not specific to podiatry practice. Given that podiatrists may work in multidisciplinary settings [31, 32], this offers the opportunity for interprofessional pre-registration education, embedding the benefits of teamwork, collaboration, family centred care and social and professional learning, whilst possibly easing pressure on curriculum resources [33, 34].

### Curriculum delivery and structure

#### Curriculum structure

The panel could not agree on how much time should be devoted to paediatric topics and when they should be introduced during the course. As such, many statements in this area did not reach agreement (42/45, 93%). This lack of agreement could reflect the background of the Delphi panel, with participants leaning more to clinical practice than academia, but is also likely to be complicated by factors such as the varying length of pre-registration podiatry programmes (from 2-4 years), curricula organisation, availability and timing of paediatric clinical placements and timing of other curriculum topics. The panel agreed that a spiral approach to organising the pre-registration paediatric podiatry curriculum was recommended, allowing students to embed and deepen their learning by revisiting themes [35].

#### Clinical paediatric experience

Increased demand for healthcare has resulted in the need to train more health professionals, necessitating more clinical placements [36, 37]. This has been a challenge across many health professions, also reflected in this present study [36, 37]. Participants agreed that mandatory paediatric clinical placements are necessary, but unfeasible, stating that the number of placements falls short of student demand (Appendix 1, Question 68). Similarly, participants were challenged to set a minimum number of paediatric cases. However, participants agreed that there are several alternatives to clinical placements and exposure to paediatric cases including: university clinics, reflective tutorials, practical sessions with ‘real’ patients, simulated inter-disciplinary learning, practical skills sessions, symptom focussed problem based learning and tutorials focussing on case studies (Table 2). With careful planning, these may be used creatively to enhance the paediatric curriculum, especially virtual sessions which are rapidly gaining popularity and credibility in the higher education sector. Simulated learning, which has been integrated into medical curricula for several years [38], can support knowledge acquisition and confidence in learning during pre- and post-registration podiatry education [39, 40].

#### Assessment, competencies and curriculum review

The recommendations of the panel to integrate paediatrics into summative assessments that contribute to the final pre-registration award contrasts with recent findings [16]. Currently there appears to be no trend when assessing students’ understanding of paediatric presentations, suggesting that, in some instances, students may not be required to demonstrate competency in paediatrics prior to qualification [16].

### Recommendations

**Fig. 3:**
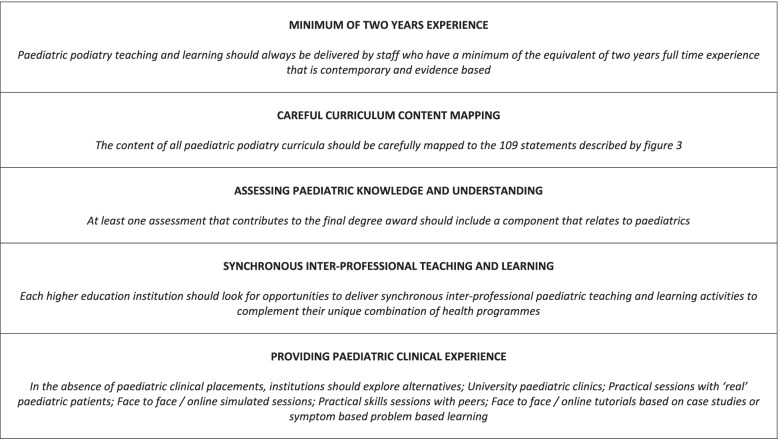
Study recommendations

Whilst post-registration paediatric podiatry benefits from clear and progressive learning and development milestones [21], pre-registration frameworks lack detail, resulting in large curricula and a likely difference in preparedness for working independently with children. If all newly registered podiatrists could access the level of support offered by the post-registration paediatric podiatry clinical framework [21] immediately upon registration, there is an argument that podiatry students need not be exposed to the level of detail described in this study. However, post-registration career pathways can be inconsistent, and the routes from registration to varied settings are poorly understood. Whilst it is hoped that all new registered podiatrists work to their clinical competencies, at present there is nothing to preclude unmonitored independent working from the day a podiatrist registers. Therefore, it is imperative that pre-registration training prepares podiatrists for the safe and effective management of children. Key recommendations from this study are presented in Fig. 3 and future action is required to embed these within local curricula. Professional bodies, higher education institutions and paediatric experts need to address whether pre-registration frameworks should be more explicit.

### Limitations

Study limitations relate to design, sample size, and panel composition. Expert consensus is accepted as the lowest level of evidence but an appropriate starting point where no other study exists. The sample size was limited by the relatively small number of podiatrists who work with children, compounded by niche characteristics required to meet the inclusion criteria. Recruitment was also impacted by the effects of the coronavirus pandemic when podiatrists were under additional strain. The composition favoured clinicians, with some limitations to academic depth.

The expertise and dedicated paediatric focused work of participants may influence their opinions on what new registered podiatrists need to understand for general day to day practice. It may be argued that this resulted in an unrealistic number of curriculum content specific statements being agreed, given that curriculum time must be balanced between different areas. Moving forwards, it seems imperative that to safeguard children’s care there must be collaboration between experts, professional bodies, clinical educators and higher education institutions. This should aim to define overarching guidelines and expectations for paediatric specific skills that UK/Australian podiatry students should meet prior to registration. Depending on the outcome, curricula documents may need to be enhanced and mentorship of newly qualified podiatrists encouraged.

## Conclusions

This research is the first to describe elements of paediatric curricula for pre-registration podiatry training. The recommendations highlight the opportunities that education providers can work towards during curriculum design. This research also provides the opportunity for benchmarking paediatric podiatry practice. Every child deserves the best health care experience and outcome, and this study provides a unique opportunity to start conversations on the future design of a binational curriculum focussing on paediatric podiatry care. It is hoped that this will inform wider, international discussions.

## Supplementary Information

**Additional file 1:** **Appendix 1.**

**Additional file 2:** **Appendix 2.**

## Data Availability

All data generated or analysed during this study are included in this published article [and its supplementary information files]. Any queries about the data or requests for further data should be sent to the corresponding author (Julie Reay).

## Abbreviations

GP: General practitioner
UK: United Kingdom
