# Supplementary material for: A step in the right direction: Delphi consensus on a UK and Australian paediatric podiatry curriculum
Source: BMC Med Educ. 2022 Feb 24;22:125. doi: 10.1186/s12909-022-03138-8 (PMC8866550; doi:10.1186/s12909-022-03138-8)
Supplement: Supplementary file 1 — Additional file 1: Appendix 1. [file 12909_2022_3138_MOESM1_ESM.docx]

**Appendix 1 – Round 1 questions**

| **Theme** | **Question number** | **Question details** |
| --- | --- | --- |
| **Participant ID number** | 1 | Please enter your participant identification code. |
| **Background of teaching staff / current modes of delivery** | 2 | In your opinion what is the minimum level of experience that teaching staff who deliver the paediatric undergraduate podiatry curriculum should have? |
|  | 3 | In your opinion what is the minimum content that should be delivered via a paediatric curriculum? (You may wish to consider whether this should be measurable objectively, for example in terms of number of paediatric patients assessed and treated or through students being assessed via a set of competencies). |
| **Curriculum content:**  **Embryology and foetal development** | 4 | Do you think that it is important to teach about embryology and foetal development? |
|  | 5 | If you answered ‘no’ to question 4, please can you explain your answer to question 4. |
|  | 6 | (If you answered ‘yes’ to question 4 please complete questions 6-9):  What would the priorities be when teaching about embryology and foetal development? |
|  | 7 | What percentage of the paediatric curriculum do you think should be devoted to embryology and foetal development? |
|  | 8 | At what point in the curriculum do you think this ought to be introduced? |
|  | 9 | What level of understanding/skills in relation to embryology and foetal development should the student be able to demonstrate as a newly qualified podiatrist? |
| **Curriculum content:**  **Ontogeny** | 10 | Do you think it is important to teach about ontogeny? |
|  | 11 | If you answered ‘no’ to question 10, please can you explain your answer to question 10. |
|  | 12 | (If you answered ‘yes’ to question 10 please complete questions 12-15):  What would the priorities be when teaching about ontogeny? (You may wish to consider the following areas; normal values, what is considered to be atypical, assessment, treatment PLEASE NOTE THAT THIS LIST IS NOT EXHAUSTIVE). |
|  | 13 | What percentage of the paediatric curriculum do you think should be devoted to ontogeny? |
|  | 14 | At what point in the curriculum do you think ontogeny ought to be introduced? |
|  | 15 | What level of understanding/skills in relation to ontogeny should the student be able to demonstrate as a newly qualified podiatrist? |
| **Curriculum content:**  **Child Social and Physical Development and Milestones** | 16 | Do you think it is important to teach about child social and physical development and milestones? |
|  | 17 | If you answered ‘no’ to question 16, please can you explain your answer to question 16. |
|  | 18 | (If you answered ‘yes’ to question 16 please complete questions 18-21):  What would the priorities be when teaching about child social and physical development and milestones? |
|  | 19 | What percentage of the paediatric curriculum do you think should be devoted to child social and physical development and milestones? |
|  | 20 | At what point in the curriculum do you think this ought to be introduced? |
|  | 21 | What level of understanding/skills in relation to child social and physical development and milestones should the student be able to demonstrate as a newly qualified podiatrist? |
| **Curriculum content:**  **Public Health, prevention and health inequalities** | 22 | Do you think it is important that undergraduate podiatry students learn about public health, prevention and health inequalities in relation to children’s feet / the lower limb? |
|  | 23 | If you answered ‘no’ to question 22, please detail why. |
|  | 24 | (If you answered ‘yes’ to question 22 please complete questions 24-27):  Please detail the areas that you think are most important in relation to public health, prevention and health inequalities (Topics you may wish to consider are; skincare, foot development, appropriate footwear, when to seek health intervention, access to services, physical activity PLEASE NOTE THAT THIS LIST IS NOT EXHASUTIVE). |
|  | 25 | What percentage of the paediatric curriculum do you think should be devoted to public health, prevention and health inequalities? |
|  | 26 | At what point in the undergraduate course do you think this ought to be introduced? |
|  | 27 | What level of understanding/skills around public health, prevention and health inequalities should the student be able to demonstrate when newly qualified? |
| **Curriculum content:**  **Atypical development during childhood / conditions specific to paediatrics** | 28 | Do you think it is important to teach about atypical development during childhood and health conditions specific to paediatrics? |
|  | 29 | If you answered ‘no’ to question 28, please detail why. |
|  | 30 | (If you answered ‘yes’ to question 28 please complete questions 30-33):  What would the priorities be with regards to atypical development / health conditions specific to paediatrics? (You may wish to consider the following areas; reasons behind atypical development, paediatric health conditions (including orthopaedic and neurological) presentation/assessment/treatment for children with atypical development / paediatric health conditions, differential diagnosis, roles within the multidisciplinary team, referral to other members of the multidisciplinary team PLEASE NOTE THAT THIS LIST IS NOT EXHAUSTIVE). |
|  | 31 | What percentage of the paediatric curriculum do you think should be devoted to atypical development / paediatric health conditions? |
|  | 32 | At what point in the curriculum do you think this ought to be introduced? |
|  | 33 | What level of understanding/skills in relation to atypical development / paediatric health conditions should the student be able to demonstrate as a newly qualified podiatrist? |
| **Curriculum content:**  **Interacting with children and parents / carers** | 34 | What do you think is the best method of supporting students to develop the professional communication skills suited to children and their families/ carers? |
|  | 35 | Please suggest the areas relating to the topic ‘interacting with children and parents / carers’ that you think are most important (Topics you may wish to consider are; communication with age appropriate children, communication with children who have a learning disability, communication with parents/carers, motivating younger children through play or distraction, how to talk to children about pain, consideration of the patient experience through the eyes of the child PLEASE NOTE THAT THIS LIST IS NOT EXHAUSTIVE). |
|  | 36 | What percentage of the paediatric curriculum do you think should be devoted to professional communication skills? |
|  | 37 | At what point in the curriculum do you think that teaching and learning on professional communication skills should be introduced? |
|  | 38 | Which skills from this area should the student be able to demonstrate as a newly qualified podiatrist? |
| **Curriculum content:**  **Child protection / health and safety** | 39 | Do you think there should be a different focus on paediatric safeguarding to adult safeguarding which forms a separate area of the paediatric curriculum? |
|  | 40 | If you answered ‘no’ to question 39, please detail why. |
|  | 41 | (If you answered ‘yes’ to question 39 please complete questions 41-43):  How much time in the curriculum do you think should be devoted to paediatric safeguarding? |
|  | 42 | At what point in the undergraduate course do you think the topic of child safeguarding should be introduced? |
|  | 43 | Which key areas about safeguarding should the student understand as a newly qualified podiatrist? |
|  | 44 | Do you feel that it is important to include paediatric basic life support in a paediatric curriculum? |
|  | 45 | If you answered ‘no’ to question 44, please detail why. |
|  | 46 | (If you answered ‘yes’ to question 44 please complete question 46):  At what point in the undergraduate curriculum course do you think that Paediatric Basic Life Support ought to be introduced? |
|  | 47 | Are there any other areas relating to child protection and health and safety that you feel should be included in a paediatric podiatry curriculum? |
| **Curriculum content: Assessment of the child** | 48 | What would you include specifically in the learning about paediatric assessment? (examples of suggestions could be age appropriate normal range of motion, tests for hypermobility, specific red flag questions for children, differential diagnosis, when to refer on to other professionals, the degree of urgency with which to refer, how to ask children about their pain, family history PLEASE NOTE THAT THIS LIST IS NOT EXHAUSTIVE). |
|  | 49 | Do you feel that a specific assessment form should be used when teaching students about the paediatric assessment? |
|  | 50 | How much time in the curriculum do you think should be devoted to this area? |
|  | 51 | At what point in the undergraduate course do you think this ought to be introduced? |
|  | 52 | Which skills from this area should the student be able to demonstrate as a newly qualified podiatrist? |
| **Curriculum content:**  **A child and family centred curriculum and person centred care** | 53 | Do you feel that it is important to build the paediatric curriculum around a child and family centred approach? |
|  | 54 | If you answered ‘no’ to question 53, please detail why. |
|  | 55 | (If you answered ‘yes’ to question 53 please complete questions 55-57):  Please suggest the topics that you would include to create a child and family centred approach? (You may wish to consider: The International Classification of Functioning, exercise, wellbeing, lifestyle changes, history taking, adherence to treatment, family history PLEASE NOTE THAT THIS LIST IS NOT EXHAUSTIVE). |
|  | 56 | How much time in the curriculum do you think should be devoted to this area? |
|  | 57 | At what point in the undergraduate course do you think this ought to be introduced? |
| **Curriculum content:**  **Paediatric outcome measures** | 58 | Do you feel that it is important to discuss child specific outcome measures? |
|  | 59 | If you answered ‘no’ to question 58, please detail why. |
|  | 60 | (If you answered ‘yes’ to question 58 please complete questions 60-64):  Do you feel that paediatric outcome measures should be specific to the impairment being treated, holistic or a combination of both? |
|  | 61 | What outcome measures would you expect students to be familiar with? |
|  | 62 | How much time do you think should be devoted to learning about paediatric outcome measures? |
|  | 63 | At what point in the undergraduate course do you think this ought to be introduced? |
|  | 64 | Which skills from this area should the student be able to demonstrate as a newly qualified podiatrist? |
| **Curriculum content**  **Other suggestions** | 65 | In addition to the topics discussed so far in this survey do you think that anything else should be included specifically in the paediatric curriculum? (Please specify) |
| **Curriculum delivery and structure** | 66 | Whilst there are different methods of teaching delivery, which do you feel are most suited for delivering the paediatric curriculum and why? (You may wish to consider the following; face to face lectures, face to face tutorials, distance based lectures in real time, online recorded lectures, online tutorials PLEASE NOTE THAT THIS LIST IS NOT EXHAUSTIVE). |
|  | 67 | Do you feel that a mandatory paediatric clinical placement (either in-house or external to the university) is necessary? |
|  | 68 | Do you feel that a mandatory clinical placement is feasible? |
|  | 69 | If not, please can you explain why. |
|  | 70 | (If you answered ‘yes’ to question 67 please complete questions 70-72):  How many hours (across the degree) do you feel is a realistic minimum standard for a paediatric clinical placement? |
|  | 71 | Not all students gain clinical experience across the array of paediatric presentations. What are the essential conditions that undergraduate students should be introduced to? |
|  | 72 | What do you think is the best method to deliver this content? (You may wish to consider the following; case studies, problem-based tutorial sessions, group feedback following clinical placements, teaching sessions focussed around clinical presentations, for example swelling, utilising a problem based approach to identify all of the possible conditions underpinning the presentation PLEASE NOTE THAT THIS LIST IS NOT EXHAUSTIVE). |
|  | 73 | Curricula can be organised in different ways. One method is to organise learning materials into self-contained modules and another is to adopt a spiral curriculum whereby information is revisited incrementally throughout the degree, allowing students to enhance their depth of learning over time. Do you have any thoughts on which approach you feel is more suited to paediatrics? (please explain why). |
| **Curriculum review** | 74 | How often do you think a paediatric curriculum should be updated? |
|  | 75 | Who do you think should have overall responsibility for updating the paediatric curriculum? |
