# Supplementary material for: A step in the right direction: Delphi consensus on a UK and Australian paediatric podiatry curriculum
Source: BMC Med Educ. 2022 Feb 24;22:125. doi: 10.1186/s12909-022-03138-8 (PMC8866550; doi:10.1186/s12909-022-03138-8)
Supplement: Supplementary file 2 — Additional file 2: Appendix 2. [file 12909_2022_3138_MOESM2_ESM.docx]

**Appendix 2 – Status of all survey statements**

| **THEME** | **STATEMENT** | **ACCEPTED**  (Percentage consensus / agreement) | **REJECTED**  (Percentage agreement) | **ROUND** |
| --- | --- | --- | --- | --- |
| **BACKGROUND OF TEACHING STAFF / CURRENT MODES OF DELIVERY** | **A lecturer who delivers the paediatric undergraduate podiatry curriculum should have the following experience? (Feel free to tick more than one box):** |  |  |  |
|  | - Minimum of 12 months working in a multidisciplinary team |  | 17% | 2 |
|  | - Minimum of 2 years post graduate paediatric clinical practice experience | 92% |  | 3 |
|  | - Minimum of 5 years post graduate paediatric clinical practice experience |  | 42% | 2 |
|  | - Minimum experience should be adjusted according to part time hours and time spent with the paediatric population |  | 33% | 2 |
|  | - Postgraduate qualification (for example equivalent to one Masters degree module) |  | 33% | 2 |
|  | - Postgraduate degree such as a Masters degree or PhD (completed or working towards) |  | 25% | 2 |

| **THEME** | **STATEMENT** | **ACCEPTED**  (Percentage consensus / agreement) | **REJECTED**  (Percentage agreement) | **ROUND** |
| --- | --- | --- | --- | --- |
| **CURRICULUM CONTENT:**  **Embryology and foetal development** | It is important to include the topic of ‘embryology’ and ‘foetal development’ in a paediatric curriculum | 92% |  | 1 |
|  | **The following are priority areas to consider when teaching about ‘embryology and foetal development’:** |  |  |  |
|  | - An overview of the typical stages of embryonic and foetal development, and an understanding of the common mechanisms that disrupt this (and their sequalae) | 75% |  | 2 |
|  | - Typical neuromusculoskeletal development of the trunk, lower limb and foot in utero and the consequences of atypical development | 92% |  | 2 |
|  | - The consequences of a premature birth | 100% |  | 2 |
| **CURRICULUM DELIVERY AND STRUCTURE: Embryology and foetal development** | **What percentage of the paediatric curriculum do you think should be devoted to studying ‘embryology and foetal development’? (Please do not tick more than one box)** |  |  |  |
|  | - 0-10% | 75% |  | 2 |
|  | - 11-20% |  | 8% | 2 |
|  | - Decided locally by each higher education institution |  | 17% | 2 |
|  | **At what point in the curriculum do you think the topic of ‘embryology and foetal development’ should be introduced? (Please do not tick more than one box)** |  |  |  |
|  | - First year |  | 42% | 2 |
|  | - Middle year(s) |  | 17% | 2 |
|  | - Final year |  | 0% | 2 |
|  | - This should be decided specific to each higher education institution |  | 33% | 2 |
|  | - This should be decided nationally by a panel with expert experience in Podiatry higher education |  | 8% | 2 |

| **THEME** | **STATEMENT** | **ACCEPTED**  (Percentage consensus / agreement) | **REJECTED**  (Percentage agreement) | **ROUND** |
| --- | --- | --- | --- | --- |
| **CURRICULUM CONTENT:**  **Ontogeny** | It is important to include the topic of ‘ontogeny’ in a paediatric curriculum (92% agreement) | 92% |  | 1 |
|  | **The following are priority areas to consider when teaching about ‘ontogeny’:** |  |  |  |
|  | - Typical development of the neuromusculoskeletal systems from birth to maturity (with a specific focus on the trunk and lower limbs), and an understanding of the common mechanisms that disrupt this (and their sequalae) | 92% |  | 2 |
|  | - Typical changes to lower limb joint range of motion (including muscle lengths) throughout childhood | 92% |  | 2 |
|  | - Typical changes to lower limb alignment and torsions (for example femoral anteversion, tibial torsion, genu valgum) throughout childhood | 100% |  | 2 |
|  | - Typical changes to foot morphology and structure(s) in early childhood | 100% |  | 2 |
|  | - Typical development of gait from infancy to maturity | 100% |  | 2 |
| **CURRICULUM DELIVERY AND STRUCTURE: Ontogeny** | **What percentage of the paediatric curriculum do you think should be devoted to studying ‘ontogeny’? (Please do not tick more than one box)** |  |  |  |
|  | - 0-10% |  | 17% | 2 |
|  | - 11-20% | 75% |  | 3 |
|  | - 21-30% |  | 25% | 2 |
|  | - 31-40% |  | 8% | 2 |
|  | - Decided locally by each higher education institution |  | 8% | 2 |
|  | **At what point in the curriculum do you think the topic of ‘ontogeny’ should be introduced? (Please do not tick more than one box)** |  |  |  |
|  | First year |  | 25% | 2 |
|  | Middle year(s) |  | 33% | 2 |
|  | Final year |  | 0% | 2 |
|  | This should be decided specific to each higher education institution |  | 33% | 2 |
|  | This should be decided nationally by a panel with expert experience in Podiatry higher education |  | 8% | 2 |

| **THEME** | **STATEMENT** | **ACCEPTED**  (Percentage consensus / agreement) | **REJECTED**  (Percentage agreement) | **ROUND** |
| --- | --- | --- | --- | --- |
| **CURRICULUM CONTENT:**  **Child social and physical development and milestones** | It is important to include the topic of ‘child social and physical development and milestones’ in a paediatric curriculum | 100% |  | 1 |
|  | **The following are priority areas to consider when teaching about ‘child social and physical development and milestones’** |  |  |  |
|  | - Gross motor milestones (for example sitting, walking and jumping) including an understanding of the typical movement patterns associated with each milestone (for example when to expect asymmetries / unilateral movements) | 84% |  | 2 |
|  | - Other developmental milestones (for example fine motor, language, communication, behaviour) | 84% |  | 2 |
|  | - The relationship between different milestones (for example fine and gross motor, gross motor and language) | 100% |  | 2 |
|  | - How social circumstances and hobbies/sporting activities affect typical development | 84% |  | 2 |
|  | - Recognising mildly delayed and gross atypical milestones and how to address concerns | 75% |  | 2 |
|  | - The importance of using trusted evidence based sources to define milestones | 75% |  | 2 |
| **CURRICULUM DELIVERY AND STRUCTURE: Child social and physical development and milestones** | **What percentage of the paediatric curriculum do you think should be devoted to studying ‘child social and physical development and milestones’? (Please do not tick more than one box)** |  |  |  |
|  | - Between 0-10% |  | 33% | 2 |
|  | - Between 11-20% |  | 33% | 2 |
|  | - Between 21-30% |  | 25% | 2 |
|  | - Decided locally by each higher education institution |  | 8% | 2 |
|  | **At what point in the curriculum do you think the topic of ‘child social and physical development milestones’ should be introduced? (Please do not tick more than one box)** |  |  |  |
|  | - First year |  | 0% | 2 |
|  | - Middle year(s) |  | 42% | 2 |
|  | - Final year |  | 8% | 2 |
|  | - This should be decided specific to each higher education institution |  | 33% | 2 |
|  | - This should be decided nationally by a panel with expert experience in Podiatry higher education |  | 17% | 2 |

| **THEME** | **STATEMENT** | **ACCEPTED**  (Percentage consensus / agreement) | **REJECTED**  (Percentage agreement) | **ROUND** |
| --- | --- | --- | --- | --- |
| **CURRICULUM CONTENT:**  **Public health, prevention and health inequalities** | It is important to include the topic of ‘public health, prevention and health inequalities’ in a paediatric curriculum | 100% |  | 1 |
|  | **The following are priority areas to consider when teaching about ‘public health, prevention and health inequalities’:** |  |  |  |
|  | - The impact of physical activity (including potential adverse effects) on wellbeing, physical and mental health | 92% |  | 3 |
|  | - The impact of diet and obesity on paediatric wellbeing, physical and mental health | 92% |  | 3 |
|  | - The importance of sleep to paediatric wellbeing, physical and mental health | 92% |  | 3 |
|  | - The impact of smoking and alcohol on wellbeing, physical and mental health |  | 67% | 4 |
|  | - The importance of paediatric skin and nail care | 75% |  | 2 |
|  | - Appropriate children’s footwear and the importance on typical development | 92% |  | 2 |
|  | - The importance to the child, family and society of health prevention and limiting co-morbidities during childhood | 84% |  | 2 |
|  | - Appropriate paediatric podiatry referrals | 100% |  | 2 |
|  | - Signposting children and parents/carers to appropriate evidence based guidance relating to typical and atypical development (for example normal foot development, milestones, ‘flat feet’, toe walking), when to access paediatric podiatry services and instances when treatment is not required | 100% |  | 2 |
|  | - Barriers to accessing paediatric services and their effect on paediatric health. Methods of promoting health equality in areas of deprivation. | 92% |  | 2 |
|  | - Paediatric health concerns that relate to specific cultures and populations | 92% |  | 2 |
|  | - Making clear links to public health and lifestyle topics in management plans | 84% |  | 3 |
|  | - An awareness that early recognition of health issues may improve outcomes | 92% |  | 3 |
| **CURRICULUM DELIVERY AND STRUCTURE: Public health, prevention and health inequalities** | **What percentage of the paediatric curriculum do you think should be devoted to studying ‘public health, prevention and health inequalities’? (Please do not tick more than one box)** |  |  |  |
|  | - Between 0-10% | 84% |  | 3 |
|  | - Between 11-20% |  | 33% | 2 |
|  | - Between 21-30% |  | 0% | 2 |
|  | - Decided locally by each higher education institution |  | 8% | 2 |

| **THEME** | **STATEMENT** | **ACCEPTED**  (Percentage consensus / agreement) | **REJECTED**  (Percentage agreement) | **ROUND** |
| --- | --- | --- | --- | --- |
| **CURRICULUM DELIVERY AND STRUCTURE: Public health, prevention and health inequalities** | **At what point in the curriculum do you think the topic of ‘public health, prevention and health inequalities’ should be introduced? (Please do not tick more than one box)** |  |  |  |
|  | - First year |  | 0% | 2 |
|  | - Middle year(s) |  | 25% | 2 |
|  | - Final year |  | 25% | 2 |
|  | - This should be decided specific to each higher education institution |  | 33% | 2 |
|  | - This should be decided nationally by a panel with expert experience in Podiatry higher education |  | 17% | 2 |

| **THEME** | **STATEMENT** | | **ACCEPTED**  (Percentage consensus / agreement) | **REJECTED**  (Percentage agreement) | **ROUND** |
| --- | --- | --- | --- | --- | --- |
| **CURRICULUM CONTENT:**  **Atypical development during childhood / conditions specific to paediatrics** | | It is important to include the topic of ‘atypical development during childhood / conditions specific to paediatrics’ in a paediatric curriculum | 100% |  | 1 |
|  |  | **The following are priority areas to consider when teaching about ‘atypical development during childhood / conditions specific to paediatrics’:** |  |  |  |
|  |  | - Reasons for delayed and regressing milestones | 100% |  | 2 |
|  |  | - Understanding the aetiology, pathogenesis, epidemiology and presentation of common atypical paediatric presentations | 100% |  | 2 |
|  |  | - Understanding evidence based podiatric management and likely outcomes of common atypical paediatric presentations | 92% |  | 2 |
|  |  | - Understanding paediatric red flags and differential diagnoses for common atypical paediatric presentations | 100% |  | 2 |
|  |  | - Understanding the scope of the podiatrist and the role of other health professionals with regards to common atypical paediatric presentations | 84% |  | 2 |
|  |  | - Understanding when to refer children to other health professionals (including the degree of urgency) | 92% |  | 2 |
|  |  | - Where to search for evidence based paediatric health summaries and management guidelines | 84% |  | 2 |
|  |  | *Understanding of the aetiology, pathogenesis, epidemiology, presentation and management of the following:* |  |  |  |
|  |  | - Orthopaedic conditions of the lower limbs | 84% |  | 2 |
|  |  | - Orthopaedic conditions of the foot | 92% |  | 2 |
|  |  | - Orthopaedic conditions of the spinal column | 84% |  | 2 |
|  |  | - (More frequently encountered) Rheumatological conditions that affect childhood development (for example Juvenile Idiopathic Arthritis, connective disorders such as Ehlers Danlos and hypermobility) | 92% |  | 2 |
|  |  | - (More frequently encountered) Genetic conditions that affect global childhood development (for example Down’s Syndrome) | 100% |  | 2 |
|  |  | - (More commonly encountered) Neurological conditions that affect childhood development (for example Autism, Cerebral Palsy, Hereditary Motor and Sensory Neuropathies [CMT], Spina Bifida, Muscular Dystrophies) | 100% |  | 2 |
|  |  | - Paediatric dermatological and nail conditions (for example Epidermolysis Bullosa) | 84% |  | 2 |
|  |  | - Paediatric atypical gait | 100% |  | 2 |
|  |  | - Childhood pain (including chronic pain) | 100% |  | 2 |

| **THEME** | **STATEMENT** | **ACCEPTED**  (Percentage consensus / agreement) | **REJECTED**  (Percentage agreement) | **ROUND** |
| --- | --- | --- | --- | --- |
| **CURRICULUM DELIVERY AND STRUCTURE: Atypical development during childhood / conditions specific to paediatrics** | **What percentage of the paediatric curriculum do you think should be devoted to studying ‘atypical development during childhood / conditions specific to paediatrics’? (Please do not tick more than one box)** |  |  |  |
|  | Between 0-10% |  | 8% | 2 |
|  | Between 11-20% |  | 33% | 2 |
|  | Between 21-30% |  | 33% | 2 |
|  | Between 31-40% |  | 17% | 2 |
|  | Decided locally by each higher education institution |  | 8% | 2 |
|  | **At what point in the curriculum do you think the topic of ‘atypical development during childhood / conditions specific to paediatrics’ should be introduced? (Please do not tick more than one box)** |  |  |  |
|  | First year |  | 8% | 2 |
|  | Middle year(s) | 92% |  | 3 |
|  | Final year |  | 17% | 2 |
|  | This should be decided specific to each higher education institution |  | 17% | 2 |
|  | This should be decided nationally by a panel with expert experience in Podiatry higher education |  | 8% | 2 |

| **THEME** | **STATEMENT** | **ACCEPTED**  (Percentage consensus / agreement) | **REJECTED**  (Percentage agreement) | **ROUND** |
| --- | --- | --- | --- | --- |
| **CURRICULUM CONTENT:**  **Interacting with children and parents and carers / A child and family centred curriculum and person centred care** | Shadowing specialist paediatric podiatrists is an important method of supporting students to develop professional communication skills suited to interactions with children and parents/carers | 100% |  | 1 |
|  | It is important to build the paediatric curriculum around a child and family centred approach | 100% |  | 1 |
|  | **The following are priority areas to consider when teaching about ‘interacting with children and parents/carers’ and ‘a child and family centred curriculum and person centred care’** |  |  |  |
|  | Holistic approaches to child centred care and the principle that ‘every child and parent/carer has a voice’. | 84% |  | 2 |
|  | Methods of communicating with age appropriate children of different ages (including toddlers, young children, adolescents, elite athletes) | 92% |  | 2 |
|  | Methods of communicating with children who have atypical communication (such as speech and cognitive impairments) | 75% |  | 2 |
|  | The impact of delayed milestones and atypical development on the child and family | 100% |  | 2 |
|  | Interview techniques for different ages / situations (including methods of discussing pain) | 92% |  | 2 |
|  | Using play and distraction to facilitate podiatric assessment and management | 92% |  | 2 |
|  | Recognising the sensitivities that surround communication with children and parents/carers and the importance of an empathetic approach, especially when discussing typical development, diagnoses and prognoses. A consideration of patient experience through the child’s and parent/carer’s eyes. | 92% |  | 2 |
|  | Strategies that may be used to re-assure children and parents/carers | 92% |  | 2 |
|  | Strategies that may be used to interact with apathetic parents/carers | 92% |  | 2 |
|  | Understanding the consequences of impressions made by clinicians on children and parents/carers (such as appearance, body language) | 84% |  | 2 |
|  | The importance of goal setting and different methods of devising and reviewing goals | 84% |  | 2 |
|  | Planning assessment, management and goals around the principles of biopsychosocial models (for example the International Classification of Functioning) | 84% |  | 2 |
|  | Methods of teaching home management strategies (such as exercise programs) to children and parents/carers and the importance of open discussions around adherence to management plans | 92% |  | 2 |
|  | The role of a key worker model in enabling paediatric continuity of care | 75% |  | 2 |
|  | Transition to adult services | 75% |  | 2 |

| **THEME** | **STATEMENT** | **ACCEPTED**  (Percentage consensus / agreement) | **REJECTED**  (Percentage agreement) | **ROUND** |
| --- | --- | --- | --- | --- |
| **DELIVERY AND STRUCTURE: Interacting with children and parents and carers / A child and family centred curriculum and person centred care** | **The following are suggested methods for developing students’ abilities to communicate with children and parents/carers:** |  |  |  |
|  | Facilitated practical sessions with peers | 84% |  | 2 |
|  | Facilitated reflective practice sessions | 92% |  | 2 |
|  | Independent student reflection | 100% |  | 3 |
|  | Peer supervision | 92% |  | 2 |
|  | Watching and discussing videos of recorded consultations | 84% |  | 2 |
|  | Developing communication skills via the use of lectures | 92% |  | 3 |
|  | National CPD activities (such as courses, online training) |  | 59% | 4 |
|  | Spending time in schools or with children’s sports teams | 75% |  | 3 |
|  | **What percentage of the paediatric curriculum do you think should be devoted to studying ‘interacting with children and parents / carers’ and ‘a child and family centred curriculum’? (Please do not tick more than one box)** |  |  |  |
|  | Between 0-10% |  | 42% | 2 |
|  | Between 11-20% |  | 42% | 2 |
|  | Between 21-30% |  | 8% | 2 |
|  | Decided locally by each higher education institution |  | 8% | 2 |
|  | **At what point in the curriculum do you think the topic of ‘interacting with children and parents / carers’ and ‘a child and family centred curriculum’ should be introduced? (Please do not tick more than one box)** |  |  |  |
|  | First year |  | 25% | 2 |
|  | Middle year(s) |  | 8% | 2 |
|  | Final year |  | 25% | 2 |
|  | This should be decided specific to each higher education institution |  | 33% | 2 |
|  | This should be decided nationally by a panel with expert experience in Podiatry higher education |  | 8% | 2 |

| **THEME** | **STATEMENT** | **ACCEPTED**  (Percentage consensus / agreement) | **REJECTED**  (Percentage agreement) | **ROUND** |
| --- | --- | --- | --- | --- |
| **CURRICULUM CONTENT:**  **Child protection / health and safety** | There should be a different focus on paediatric safeguarding to adult safeguarding in the podiatry undergraduate curriculum | 100% |  | 1 |
|  | It is important to include paediatric basic life support in a paediatric podiatry curriculum | 84% |  | 1 |
|  | **The following are priority areas to consider when teaching about ‘child protection’:** |  |  |  |
|  | Awareness of safeguarding acts, policies and drivers (For example ‘Every child matters’) | 92% |  | 2 |
|  | Signs of child abuse and neglect | 100% |  | 2 |
|  | Signs of fabricated illnesses | 100% |  | 2 |
|  | Appropriate professionals with whom to discuss a paediatric safeguarding concern (acknowledging that this is often governed locally) | 92% |  | 2 |
|  | How to refer paediatric safeguarding concerns (acknowledging that this is often governed locally) | 92% |  | 2 |
|  | Processes to be followed following a safeguarding referral (including information sharing and documenting safeguarding referrals) | 92% |  | 2 |
|  | The processes that apply to children who have been identified as ‘vulnerable’ | 100% |  | 2 |
|  | Mandatory and legislative safeguarding requirements as a registered Podiatrist | 84% |  | 2 |
|  | Safeguarding measures to protect podiatrists (for example lone working / children who attend appointments independently) | 92% |  | 2 |
|  | Health and safety of the clinical environment with reference to children | 92% |  | 2 |
| **CURRICULUM DELIVERY AND STRUCTURE: Child protection / health and safety** | **What percentage of the paediatric curriculum do you think should be devoted to studying ‘paediatric safeguarding’? (Please do not tick more than one box)** |  |  |  |
|  | Between 0-5% | 75% |  | 2 |
|  | Between 6-10% |  | 17% | 2 |
|  | Between 11-15% |  | 8% | 2 |
|  | Decided locally by each higher education institution |  | 0% | 2 |
|  | **At what point in the curriculum do you think the topic of ‘paediatric safeguarding’ should be introduced? (Please do not tick more than one box)** |  |  |  |
|  | First year |  | 0% | 2 |
|  | Middle year(s) |  | 25% | 2 |
|  | Final year |  | 25% | 2 |
|  | This should be decided specific to each higher education institution |  | 33% | 2 |
|  | This should be decided nationally by a panel with expert experience in Podiatry higher education |  | 17% | 2 |

| **THEME** | **STATEMENT** | **ACCEPTED**  (Percentage consensus / agreement) | **REJECTED**  (Percentage agreement) | **ROUND** |
| --- | --- | --- | --- | --- |
| **CURRICULUM DELIVERY AND STRUCTURE: Child protection / health and safety** | **At what point in the curriculum do you think the topic of ‘paediatric basic life support’ should be introduced? (Please do not tick more than one box)** |  |  |  |
|  | First year |  | 33% | 2 |
|  | Middle year(s) |  | 8% | 2 |
|  | Final year |  | 17% | 2 |
|  | This should be decided specific to each higher education institution |  | 33% | 2 |
|  | This should be decided nationally by a panel with expert experience in Podiatry higher education |  | 8% | 2 |

| **THEME** | **STATEMENT** | **ACCEPTED**  (Percentage consensus / agreement) | **REJECTED**  (Percentage agreement) | **ROUND** |
| --- | --- | --- | --- | --- |
| **CURRICULUM CONTENT:**  **Assessment of the child** | Students should use a specific paediatric assessment form when learning how to assess children in the clinical environment | 100% |  | 1 |
|  | **The following are priority areas to consider when teaching about subjective and objective ‘assessment of the child’:** |  |  |  |
|  | Paediatric subjective assessment / history (including birth history, social circumstances, developmental milestones, family history, pain and paediatric red flags) | 100% |  | 2 |
|  | An understanding of the common assessment tools that exist and the factors to consider when choosing such tools | 84% |  | 2 |
|  | Exposure to common assessment tools | 84% |  | 2 |
|  | Appropriate places to search for paediatric guidelines to support assessment and published assessment tools | 75% |  | 2 |
|  | An understanding of how to interpret additional investigations instigated by other health professionals (for example blood tests, radiology results) | 84% |  | 2 |
|  | Objective assessment of paediatric red flags | 92% |  | 2 |
|  | Objective paediatric musculoskeletal assessment (including joint range, muscle lengths, quality of movement, strength, foot structure and position, lower limb alignment torsions) | 92% |  | 2 |
|  | Objective paediatric neurological assessment | 92% |  | 2 |
|  | Objective paediatric rheumatology (including hypermobility) assessment | 100% |  | 2 |
|  | Objective assessment of general developmental milestones | 100% |  | 2 |
|  | Objective assessment of gross motor milestones and function | 100% |  | 2 |
|  | Objective paediatric gait assessment | 100% |  | 2 |
|  | Objective paediatric dermatology assessment | 92% |  | 2 |
|  | Objective paediatric vascular assessment | 84% |  | 2 |
|  | Objective assessment of typically developing / borderline atypical paediatric foot postures at different stages of development | 100% |  | 2 |
| **CURRICULUM DELIVERY AND STRUCTURE: Assessment of the child** | **What percentage of the paediatric curriculum do you think should be devoted to studying ‘assessment of the child’? (Please do not tick more than one box)** |  |  |  |
|  | Between 0-10% |  | 8% | 2 |
|  | Between 11-20% |  | 33% | 2 |
|  | Between 21-30% | 100% |  | 3 |
|  | Between 31-40% |  | 0% | 2 |
|  | Decided locally by each higher education institution |  | 8% | 2 |

| **THEME** | **STATEMENT** | **ACCEPTED**  (Percentage consensus / agreement) | **REJECTED**  (Percentage agreement) | **ROUND** |
| --- | --- | --- | --- | --- |
| **CURRICULUM DELIVERY AND STRUCTURE: Assessment of the child** | **At what point in the curriculum do you think the topic of ‘assessment of the child’ should be introduced? (Please do not tick more than one box)** |  |  |  |
|  | First year |  | 17% | 2 |
|  | Middle year(s) | 75% |  | 3 |
|  | Final year |  | 0% | 2 |
|  | This should be decided specific to each higher education institution |  | 25% | 2 |
|  | This should be decided nationally by a panel with expert experience in Podiatry higher education |  | 8% | 2 |

| **THEME** | **STATEMENT** | **ACCEPTED**  (Percentage consensus / agreement) | **REJECTED**  (Percentage agreement) | **ROUND** |
| --- | --- | --- | --- | --- |
| **CURRICULUM CONTENT:**  **Paediatric outcome measures** | It is important to discuss child specific outcome measures | 100% |  | 1 |
|  | Paediatric outcome measures should be both holistic and specific to the impairment being treated | 100% |  | 1 |
|  | **The following are priority areas to consider when teaching about ‘paediatric outcome measures’** |  |  |  |
|  | The importance of paediatric outcome measures. An understanding that an array of published paediatric outcome measures exists and the factors to consider when choosing such measures. | 100% |  | 2 |
|  | Appropriate places to search for paediatric outcome measures | 84% |  | 3 |
|  | Exposure to common paediatric outcome measures | 100% |  | 2 |
|  | The importance of using holistic / patient reported outcome measures (linking to principles of ICF) | 92% |  | 2 |
|  | Outcome measures specific to paediatric musculoskeletal conditions | 92% |  | 2 |
|  | Outcome measures specific to paediatric neurological conditions | 92% |  | 2 |
|  | Outcome measures specific to paediatric rheumatology / hypermobility | 92% |  | 2 |
|  | Outcome measures specific to paediatric general development | 92% |  | 2 |
|  | Outcome measures specific to paediatric gross motor milestones and function | 92% |  | 2 |
|  | Outcome measures specific to paediatric pain | 92% |  | 2 |
|  | Outcome measures specific to paediatric gait | 92% |  | 2 |
|  | Outcome measures specific to paediatric balance | 92% |  | 2 |
|  | Outcome measures specific to paediatric dermatology | 84% |  | 2 |
|  | Outcome measures specific to paediatric vascular conditions | 75% |  | 2 |
|  | The relationship between outcome measures and commissioning/funding for health services or NDIS participants | 92% |  | 3 |
| **CURRICULUM DELIVERY AND STRUCTURE: Paediatric outcome measures** | **What percentage of the paediatric curriculum do you think should be devoted to studying ‘paediatric outcome measures’? (Please do not tick more than one box)** |  |  |  |
|  | Between 0-5% | 75% |  | 3 |
|  | Between 6-10% |  | 33% | 2 |
|  | Between 11-20% |  | 8% | 2 |
|  | Decided locally by each higher education institution |  | 8% | 2 |
|  | **At what point in the curriculum do you think the topic of ‘paediatric outcome measures’ should be introduced? (Please do not tick more than one box)** |  |  |  |
|  | First year |  | 0% | 2 |
|  | Middle year(s) |  | 25% | 2 |
|  | Final year | 84% |  | 3 |
|  | This should be decided specific to each higher education institution |  | 17% | 2 |
|  | This should be decided nationally by a panel with expert experience in Podiatry higher education |  | 8% | 2 |
| **THEME** | **STATEMENT** | **ACCEPTED**  (Percentage consensus / agreement) | **REJECTED**  (Percentage agreement) | **ROUND** |
| **CURRICULUM DELIVERY AND STRUCTURE** | A mandatory paediatric clinical placement (either in-house or external to the university) is necessary | 92% |  | 1 |
|  | A spiral approach to organising the undergraduate paediatric podiatry curriculum appears to be more suitable than self-contained modules | 77% |  | 1 |
|  | **Whilst consensus was achieved in round one that ‘A mandatory paediatric clinical placement is necessary’ there was only 62% agreement that this is feasible. The reason underpinning this lack of consensus is the limited number of paediatric placements to number of podiatry students. In the absence of clinical placements, do you agree that it is acceptable to substitute clinical placements with other forms of ‘real life’ paediatric podiatry clinical experience?** |  |  |  |
|  | Yes | 92% |  | 2 |
|  | No |  | 8% | 2 |
|  | **In the absence of clinical placements, the following are suitable alternatives to support ‘real life’ paediatric clinical experience** |  |  |  |
|  | Face to face / web based tutorial sessions focussed on case studies based on ‘real’ patients | 92% |  | 2 |
|  | Symptom based problem solving teaching sessions | 75% |  | 2 |
|  | Face to face practical ‘skills’ based sessions with peers | 75% |  | 2 |
|  | Simulated inter-disciplinary learning sessions | 75% |  | 3 |
|  | Practical sessions with ‘real’ patients | 92% |  | 2 |
|  | Reflective tutorials following clinical placements | 92% |  | 3 |
|  | University clinics | 75% |  | 2 |
|  | There are no suitable alternatives to clinical placements |  | 25% | 2 |
|  | **Do you agree that there should be a designated number of mandatory hours attached to paediatric clinical experience (relating to clinical placement / any agreed alternatives according to the question above)?** |  |  |  |
|  | Yes |  | 25% | 2 |
|  | No |  | 8% | 2 |
|  | Instead of number of hours, this should be measured by number of patient cases | 92% |  | 3 |

| **THEME** | **STATEMENT** | **ACCEPTED**  (Percentage consensus / agreement) | **REJECTED**  (Percentage agreement) | **ROUND** |
| --- | --- | --- | --- | --- |
| **CURRICULUM DELIVERY AND STRUCTURE** | **How many hours / patient cases should be devoted to paediatric clinical experience (clinical placement / any agreed alternatives)? (Please do not tick more than one box)** |  |  |  |
|  | 25 hours |  | 0% | 2 |
|  | 50 hours |  | 17% | 2 |
|  | 75 hours |  | 0% | 2 |
|  | 100 hours |  | 0% | 2 |
|  | 101-150 hours |  | 0% | 2 |
|  | 151-200 hours |  | 0% | 2 |
|  | 1-10 clinical cases |  | 8% | 2 |
|  | 11-20 clinical cases |  | 25% | 2 |
|  | More than 20 clinical cases |  | 25% | 2 |
|  | The exact amount should be decided specific to each higher education institution |  | 8% | 2 |
|  | The exact amount should be decided nationally by a panel with expert experience in Podiatry higher education |  | 17% | 2 |
|  | **Not all students gain clinical experience across the array of paediatric presentations. Do you agree that there should be a minimum set of common paediatric presentations that undergraduate students are exposed to clinically?** |  |  |  |
|  | Yes |  | 25% | 2 |
|  | No |  | 0% | 2 |
|  | No, instead it is more important to focus on the ability to assess and recognise atypical presentations |  | 17% | 2 |
|  | Yes, but this is a balance between exposure to common presentations and a focus on the ability to assess and recognise atypical presentations | 100% |  | 3 |
|  | **Which of the following do you agree should be used to measure undergraduate clinical paediatric experience? (Please feel free to select more than one option)** |  |  |  |
|  | Reflective logbook | 84% |  | 3 |
|  | Locally defined undergraduate paediatric competencies |  | 0% | 2 |
|  | Nationally defined undergraduate paediatric competencies | 100% |  | 2 |
|  | Formal assessment | 100% |  | 3 |
|  | None |  | 0% | 2 |

| **THEME** | | **STATEMENT** | **ACCEPTED**  (Percentage consensus / agreement) | | **REJECTED**  (Percentage agreement) | | **ROUND** | |  |
| --- | --- | --- | --- | --- | --- | --- | --- | --- | --- |
| **CURRICULUM DELIVERY AND STRUCTURE** | | **Students should demonstrate understanding of the following (via clinical placement / any agreed alternatives before they graduate** |  | |  | |  | |  |
|  |  | ***Typical development:*** |  | |  | |  | |  |
|  |  | Variations in femoral anteversion, genu valgum/varum, tibial torsion, tibial bowing | 92% | |  | | 2 | |  |
|  |  | A range of typically developing feet across childhood and adolescence | 84% | |  | | 2 | |  |
|  |  | Assymptomatic pes planus | 92% | |  | | 2 | |  |
|  |  | Juvenile hallux valgus, metatarsus adductus, curly toes / lesser digital deformities | 92% | |  | | 2 | |  |
|  |  | ***Foot alignment – symptomatic:*** |  | |  | |  | |  |
|  |  | Symptomatic pes planus | 92% | |  | | 2 | |  |
|  |  | Pes cavus | 92% | |  | | 2 | |  |
|  |  | ***Structural changes of the foot:*** |  | |  | |  | |  |
|  |  | Clubfoot | 92% | |  | | 2 | |  |
|  |  | Tarsal coalition | 92% | |  | | 2 | |  |
|  |  | Avascular necrosis of the bones of the foot | 92% | |  | | 2 | |  |
|  |  | Accessory bones of the foot | 92% | |  | | 2 | |  |
|  |  | ***Lower limb musculoskeletal conditions:*** |  | |  | |  | |  |
|  |  | Perthes disease and developmental hip dysplasias | 92% | |  | | 2 | |  |
|  |  | Slipped upper femoral epiphysis | 92% | |  | | 2 | |  |
|  |  | Severe leg length discrepancies | 84% | |  | | 2 | |  |
|  |  | Traction apophysitis | 92% | |  | | 2 | |  |
|  |  | Bone tumours | 92% | |  | | 2 | |  |
|  |  | ***Gait presentations / symptoms commonly assessed via gait assessment:*** |  | |  | |  | |  |
|  |  | Toe walking | 92% | |  | | 2 | |  |
|  |  | Ataxic gait | 92% | |  | | 2 | |  |
|  |  | In-toeing and out-toeing gait | 92% | |  | | 2 | |  |
|  |  | ***Pain:*** |  | |  | |  | |  |
|  |  | Acute and chronic manifestations of pain within the feet | 84% | |  | | 2 | |  |
|  |  | ‘Growing pains’ | 84% | |  | | 2 | |  |
|  |  | Chronic regional pain syndrome | 75% | |  | | 2 | |  |
|  |  | ***Skin and nails:*** |  | |  | |  | |  |
|  |  | Epidermolysis Bullosa | 84% | |  | | 2 | |  |
|  |  | Ingrown toenails | 84% | |  | | 2 | |  |
|  |  | Verrucae | 84% | |  | | 2 | |  |
| **THEME** | **STATEMENT** | | | **ACCEPTED**  (Percentage consensus / agreement) | | **REJECTED**  (Percentage agreement) | | **ROUND** | |
| **CURRICULUM DELIVERY AND STRUCTURE** | ***Neurological and developmental delay:*** | | |  | |  | |  | |
|  | Autism | | | 84% | |  | | 2 | |
|  | Cerebral Palsy | | | 84% | |  | | 2 | |
|  | Hereditary motor and sensory neuropathy (for example Charcot Marie Tooth) | | | 84% | |  | | 2 | |
|  | Developmental delay | | | 92% | |  | | 2 | |
|  | ***Systemic:*** | | |  | |  | |  | |
|  | Juvenile idiopathic arthritis | | | 92% | |  | | 2 | |
|  | Hypermobility | | | 92% | |  | | 2 | |
|  | Diabetes | | | 84% | |  | | 2 | |
|  | Arthrogryposis | | | 84% | |  | | 2 | |
|  | Mental health conditions such as anorexia and self-harm | | | 92% | |  | | 3 | |
|  | **Who do you think should have overall responsibility for designing nationally defined undergraduate paediatric competencies? (Please feel free to tick more than one box)** | | |  | |  | |  | |
|  | Podiatry university staff | | | 100% | |  | | 3 | |
|  | Paediatric podiatry special interest groups | | | 84% | |  | | 3 | |
|  | Clinical educators | | | 84% | |  | | 3 | |
|  | A group of national/international experts | | | 75% | |  | | 2 | |
|  | Input from other paediatric health disciplines | | |  | | 67% | | 4 | |
|  | N/A – I don’t agree that nationally defined paediatric competencies are required | | |  | | 0% | | 2 | |
|  | **Do you agree that the paediatric curriculum should be included in assessments that contribute to the final undergraduate award?** | | |  | |  | |  | |
|  | Yes | | | 100% | |  | | 2 | |
|  | No | | |  | | 0% | | 2 | |

| **THEME** | **STATEMENT** | **ACCEPTED**  (Percentage consensus / agreement) | **REJECTED**  (Percentage agreement) | **ROUND** |
| --- | --- | --- | --- | --- |
| **CURRICULUM REVIEW** | **How often do you think a paediatric curriculum should be evidence checked? (Please do not tick more than one box)** |  |  |  |
|  | Annually |  | 17% | 2 |
|  | Every two years |  | 33% | 2 |
|  | Every three years |  | 25% | 2 |
|  | This should be decided nationally by a panel of experts in Podiatry higher education |  | 25% | 2 |
|  | **Who do you think should have substantial input during update of the paediatric podiatry curriculum? (Please feel free to tick more than one box)** |  |  |  |
|  | Podiatry university staff | 92% |  | 2 |
|  | Paediatric podiatry special interest groups | 83% |  | 2 |
|  | Clinical educators | 75% |  | 3 |
|  | A group of national/international experts | 92% |  | 3 |
|  | Input from other paediatric health disciplines |  | 25% | 2 |
